# Rate of atrial fibrillation by Holter-Stroke Risk Analysis in undetermined TIA/rapidly improving stroke symptoms patients

**DOI:** 10.3389/fneur.2024.1353812

**Published:** 2024-04-29

**Authors:** F Cannizzaro, A Izquierdo, D Cocho

**Affiliations:** ^1^Family Medicine Department, Hospital General de Granollers, Barcelona, Spain; ^2^Neurology Department, Hospital General de Granollers, Barcelona, Spain; ^3^Faculty of Medicine and Health Sciences, Department of Medicine, Universitat Internacional de Catalunya, Barcelona, Spain

**Keywords:** undetermined TIA, rapidly improving stroke symptoms, Holter-SRA, atrial fibrillation, neurovascular

## Abstract

**Introduction:**

Holter-SRA (Stroke Risk Analysis) is an automated analysis of ECG monitoring for Atrial Fibrillation (AF) detection. The aim of this study was to evaluate the rate of AF in undetermined TIA/Rapidly improving stroke symptoms (RISS) patients.

**Methods:**

Prospective study of undetermined TIA/RISS patients who presented to the emergency department. Early vascular studies (angio CT, transthoracic echocardiography and ECG) were performed in emergency department. The Holter-SRA device was placed for 2 h and the patients were classified into: confirmed AF, high risk of AF or low risk of AF. Prolonged ambulatory monitoring (7 days) was carried out every month for patients with a high-risk pattern. The results were evaluated until definitive detection of AF or low-risk pattern. The endpoints were rate of AF and vascular recurrence at 90 days.

**Results:**

Over a period of 24 months, 83 undetermined TIA/RISS patients were enrolled. The mean age was 70 ± 10 years and 61% were men. The median ABCD^2^ score was 4 points (1–7). After 2 h of monitoring in the emergency department, AF was detected in one patient (1.2%), 51 patients with a low-risk pattern and 31 patients (37.3%) showed a high-risk pattern of AF. During the ambulatory monitoring, of the 31 patients high risk pattern patients, AF was diagnosed to 17 cases and of the 51 patients with a low-risk pattern, one case experienced a recurrent vascular due to undetected AF (1.9% false negative). Three patients (3.6%) suffered a vascular recurrence within the first 90 days, before AF diagnosis.

**Conclusions:**

In our study, AF was detected in 22.9% of the 83 patients with indeterminate TIA/RISS. Holter-SRA has allowed us to increase the detection of AF, especially those patients with a high-risk pattern in the first 3 months.

## Introduction

Atrial fibrillation (AF) is a significant risk factor for stroke, accounting for ~20–30% of all ischemic strokes (1). Early detection of AF is crucial because anticoagulation treatment can reduce the risk of stroke by 67%.

Transient ischemic attack (TIA) is often considered a minor event, but it is still a medical emergency that requires prompt evaluation and treatment. The risk of stroke after TIA during the first week is 2–10% and during the first 90 days 8–20% (2–5). This high recurrence rate could be due to unknown AF in up to 30% of cases (6, 7). Therefore, early identification and treatment of these cases is essential to prevent vascular recurrences (8–10).

Although most Stroke Units are equipped with continuous ECG monitoring (CEM), this method carries various limitations: (1) There is a limited time window available for detection of AF during hospitalization, (2) Numerous false positive alarms require verification by an expert neurologist or cardiologist, (3) It does not allow for the assessment of the risk of future AF, (4) The necessary monitoring time in TIA/Rapidly improving stroke symptoms (RISS) patients is controversial (8, 11).

The use of machine learning algorithms for AF detection may improve the accuracy and efficiency of AF diagnosis compared to traditional interpretation of ECG recordings, without the need for hospitalization of patients. The Holter-Stroke Risk Analysis (SRA) is a novel method for automated analysis of ECG monitoring, which enables both the detection of AF and the prediction of high risk of AF (12–14). It applies an automated analysis algorithm based on machine learning to identify characteristic irregularities in the R-R interval between AF episodes, absence of P-tops, and wave patterns that continuously change in shape, duration, amplitude and direction.

Our aim was to assess the rate of AF detected by Holter-SRA in undetermined TIA/RISS patients who presented to the emergency department without requiring hospitalization for continuous ECG monitoring.

## Methods

We prospectively studied all patients with suspected TIA/RISS in the emergency department from July 2020 to July 2022. TIA was defined as a transient episode of neurologic dysfunction due to focal brain or retinal ischemia without acute infarction on initial CT scan (5). MRI was performed in all patients and we considered RISS patients to be those with DWI lesion on MRI.

All patients who had sufficient clinical suspicion to warrant neurovascular imaging were eligible for inclusion in this study. The protocol was approved by the Ethics Committee, and all patients were provided written informed consent.

In the emergency department, a stroke neurologist evaluated the following assessments in all patients: ABCD^2^ score (≥60 years = 1 point; systolic blood pressure ≥ 140 mmHg or diastolic ≥ 90 mmHg = 1 point; unilateral weakness = 2 points; speech disturbance without weakness = 1 point; duration of symptoms ≥60 min = 2 points, 10–59 min = 1 point, and < 10 min = 0 points; and diabetes mellitus = 1 point), medical history, physical examination, routine blood chemistry, ECG, chest x-ray and cranial computed tomography (CT). The complete vascular study by Transthoracic echocardiography (TTE) including the left atrial size and carotid ultrasound or angio CT was performed in Emergency Department. Intracranial stenosis was diagnosed if the mean blood flow velocity was >80 cm/s, with side-to-side differences >30% and signs of disturbed flow (aliasing). Internal carotid stenosis was considered if PSV (peak systolic velocity) >125 cm/s or ICA/CCA PSV ratio >2. All cases were confirmed by CT or MRI angiography. Cervical ICA atherosclerosis was classified according to NASCET criteria as absent or mild (< 50% stenosis), moderate (50–69% stenosis), or severe (≥70% stenosis).

In order to select TIAs of undetermined cause, the TOAST criteria (15) were used: large -artery occlusive disease (LA), small-vessel disease (SV), cardioembolism (CE), other causes (OC), or undetermined cause (UND). They were further classified according to the arterial territory affected, including carotid, retinal, vertebrobasilar or an undetermined territory.

The following patients were excluded: those with a documented history of AF (either prior to admission or upon presentation in the emergency department with accompanying ECG), those with a TIA of known cause, or those who declined to participate in the current study.

Patients without intracranial stenosis, normal ICA or plaques < 50% were considered as normal vascular study. In these cases, the Holter-SRA device was placed for 2 h during their stay in the Emergency Department.

The Holter-SRA (SRAclinic^®^ Apoplex medical technologies Inc., Pirmasens, Germany) applies an automated analysis algorithm based on machine learning to identify characteristic irregularities in the R-R interval between AF episodes, absence of P-tops, and wave patterns that continuously change in shape, duration, amplitude and direction (16, 17). This information is obtained based on the recognition of repetitive patterns that are specific for the atrial remodeling associated with AF (12, 18). The SRA report provides three levels of risk: confirmed AF, low risk of AF, or high risk of AF.

Patients with a high-risk AF pattern detected after 2 h of monitoring in the Emergency Department, were discharged with the Holter-SRA in place for extended monitoring (7 days). A neurologist assessed the outcomes of the 7-day ambulatory Holter-SRA monitoring at the time of its removal. When the Holter was removed, the treating physician received the report within 5 min. In cases where a high-risk pattern persisted, additional prolonged monitoring was conducted every 30 days until a definitive detection of AF or a transition to a low-risk pattern, for a maximum period of 2 years. Prolonged ambulatory monitoring was not performed in patients with a low-risk pattern, given the high negative predictive value of the Holter-SRA in previous studies (96.2–100%) (13, 14, 19). All patients with AF were reviewed by a cardiologist within 24 h in order to confirm the diagnosis. Once the diagnosis was confirmed, the antiplatelet treatment was changed to anticoagulation at that time.

### Baseline vascular risk factors

Smoking was considered present if the patient reported smoking cigarettes during the 5 years preceding the study. Hypertension (HTA) was defined as a systolic blood pressure ≥ 140 mmHg or diastolic ≥ 90 mmHg, or current use of antihypertensive treatment. Diabetes mellitus was considered present in patients with a history of fasting blood glucose ≥ 126 mg/dL, HbA1c ≥ 6.5%, or current use of antidiabetic drugs. Hypercholesterolemia was defined as a total cholesterol level ≥ 200 mg/dL or current use of lipid-lowering agents.

### Clinical endpoints

Recurrent vascular events including stroke, recurrent TIA, myocardial infarction, or death, as well as therapeutic intervention, were recorded during a face-to-face interview at the 90-day follow-up. Recurrent TIA was defined as an additional TIA event occurring after emergency department admission. Stroke was defined as rapid onset of clinical symptoms of focal cerebral or retinal function disturbance, with an apparent vascular cause and acute infarction on neuroimaging.

### Statistical analysis

Statistical analysis was performed using the SPSS software, version 19.0 (IBM, Chicago, IL). Continuous variables that followed a normal distribution were summarized by the number of valid cases (N), mean, and standard deviation (SD). For non-normally distributed variables, the median and interquartile ranges were used. Categorical variables were described by the number of valid cases and the associated percentage for each category. Odds ratios (OR) for variables associated with AF were determined using multivariable logistic regression analysis, adjusted for variables with a *p*-value of < 0.1 in the univariate analysis. A *p*-value of < 0.05 was considered statistically significant.

## Results

**Figure 1:**
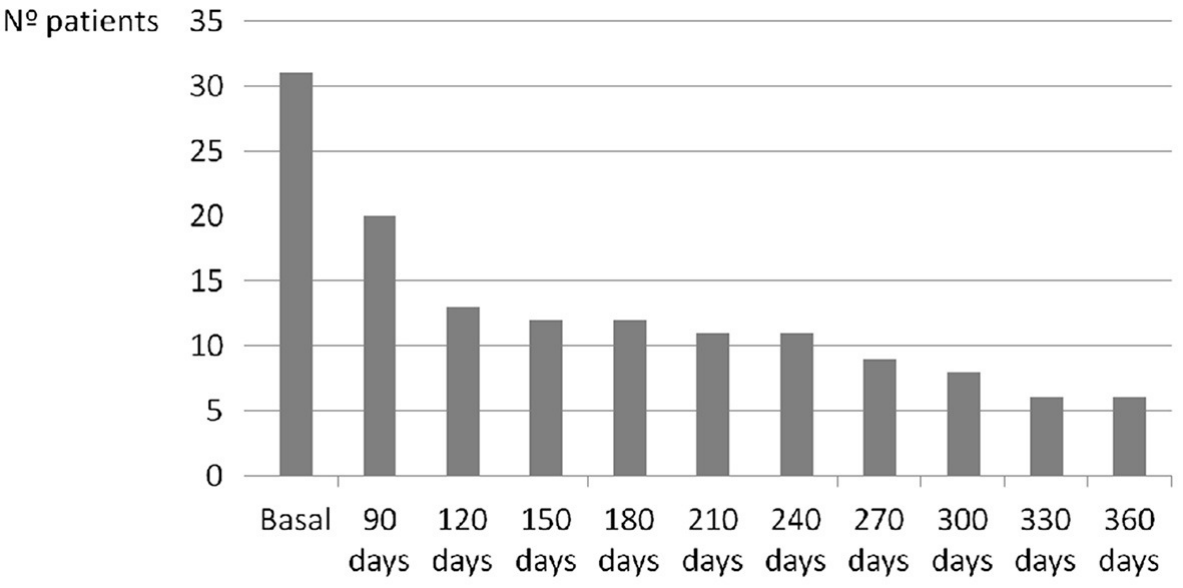
Evolution over time in the number of patients with a high-risk pattern in the SRA Holter.

A total of 87 patients were evaluated over a period of 24 months. Four patients were excluded from the study due to mimic stroke (one with multiple sclerosis, two with seizures, and one with migraine), resulting a final sample of 83 patients (**Figure 3**). The median ABCD^2^ score was 4 points (range: 1–7), and 61% of the participants were male with a mean age of 70 ± 10 years. Vascular risk factors were present in 85% of the cases, and 1% of the patients had experienced a retinal TIA. MRI was performed with a mean delay of 15 ± 10 days, and acute infarcts that were not visible on the initial CT scan were detected in 18% of the patients. These cases were considered RISS patients (Table 1). After 2 h of monitoring in the emergency department, AF was detected in one patient (1.2%), 51 patients with a low-risk pattern and 31 patients (37.3%) showed a high-risk pattern of AF. In those cases, prolonged ambulatory monitoring (7 days) was performed, with a median duration of 176 h (range: 122–2,080). AF was detected in 18 patients (21.6%), but after review by the cardiologist, AF was not confirmed in one case (5.5% false-positive). At 360 days of follow-up, six patients (7.2%) continued to present a high-risk pattern of AF (Figure 1).

**Table 1:** Baseline characteristics of study population.

	**Undetermined TIA/RISS and patients (*N* = 83)**
Age (mean, SD)	70 ± 10
Male	61%
Hypertension	67%
Diabetes mellitus	32%
Smoking	23%
Coronary heart disease	12%
Hypercholesterolemia	50%
Previous stroke	19%
**Clinical features**	
Carotid TIA	54.2%
Retinal	1.2%
Vertebrobasilar	13.3%
Undetermined	31.3%
ABCD^2^ median, (IQR)	4 (1–7)
Acute infarct on MRI	17.6%
Lacunar infarct	6.8%
Non-lacunar infarct	9.5%
Multiples territories	1.4%

IQR, interquartile range; RISS, rapidly improving stroke symptoms.

Regarding the 51 patients with a low-risk pattern of AF after 2 h of monitoring in the emergency department, one case experienced a recurrent vascular event at 62nd day due to undetected AF (1.9% false negative). After excluding the false-positive case and including the false-negative case and the patient detected after 2 h of monitoring, the final number of AF detected in our study was 19 cases (22.9%).

**Figure 2:**
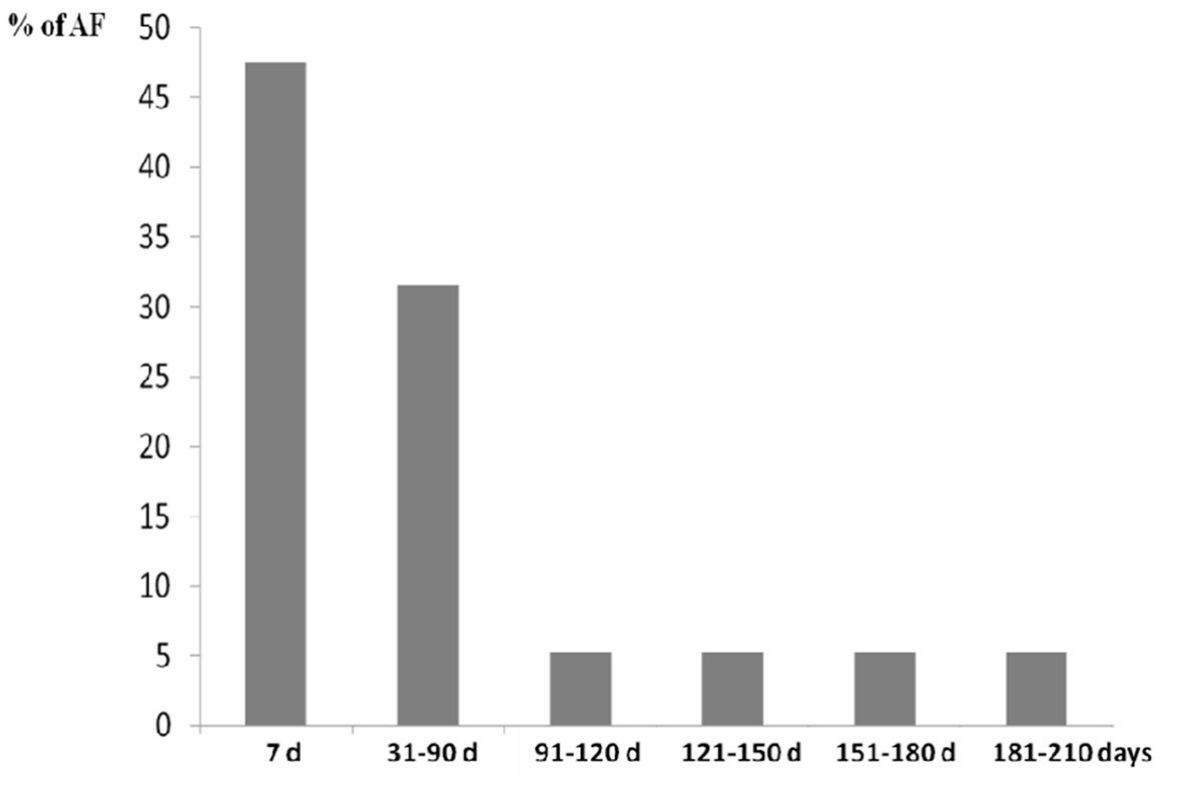
Percentage of atrial fibrillation detected over time in our study population.

According to detection time of 19 cases with AF after TIA/RISS patients, nine cases (47.4%) were detected in the first 7 days, six cases (31.6%) between 31 and 90 days, one case (5.3 %) between 91 and 120 days, one case (5.3%) between 121 and 150 days, one case (5.3%) between 151 and 180 days, and one case (5.3%) between 181 and 210 days (Figure 2).

During the first 90 days of follow-up, 3 patients (3.6%) suffered vascular recurrence, all due to undetected AF. One patient had vascular recurrence at 9^th^ day, detecting AF as etiology at 34^th^ day. Another patient suffered a new stroke at 62^nd^ day with AF detected during this second hospitalization, and another patient at 85 ^th^ day detecting AF as cause at 126^th^ day. The SRA- Holter showed a low rate of false positives (5.5%) and negatives (1.9%). Among patients with a high-risk pattern, AF was detected in 54.8 % of cases, with a sensitivity of 94%, specificity of 78%, positive predictive value (PPV) of 54%, and negative predictive value (NPV) of 98%. The univariate analysis identified the size of the left atrium (p=0.013) and age (p=0.1) as predictors of AF. Multivariate logistic regression identified only the size of the left atrium as an independent predictor of AF (Table 2).

**Table 2:** Predictors of atrial fibrillation detected in the univariate and multivariable analysis.

	**Univariate**	**Multivariate**		
		***P*** **-value**	**OR**	**95% IC**
Size left atrium	*P* = 0.013	0.04	1.12	(1.00–1.24)
Age	*P* = 0.1	0.15	1.05	(0.98–1.12)

## Discussion

In the present study, we found that 22.9% of patients with undetermined TIA/RISS had unknown AF, and all recurrences within the first 90 days were attributed to this factor. The presence of a high-risk pattern in Holter-SRA was associated with the detection of AF in 54.8% of these cases, and the only independent predictor of AF was the size of the left atrium (OR 1.12) (Table 2). According to previous studies, the size, function and anatomy of the left atrium influence the risk of stroke. Consideration of these additional factors could help when selecting patients for more intensive monitoring (20). In stroke patients, cardiac monitoring is an essential part of the evaluation to exclude AF. Most Stroke Units are equipped with ECG equipment, enabling standard continuous ECG monitoring (CEM) during the length of stay of the acute ischemic stroke patients. Depending on the settings, CEM may be highly sensitive for signs of AF but generates continuous and numerous false positive alarms leading to loss of productivity by the Stroke Unit nurses. The Stroke Risk Analysis (SRA) is a new approach for automated analysis of CEM, enabling both the detection of AF as well as the prediction of the high risk of AF (14). Currently, SRA provides a significant improvement in the detection of patients with AF compared to continuous ECG monitoring (CEM) in the stroke unit, increasing the new cases by more than 2-fold and achieving a 3-times faster diagnosis (14). In patients with sinus rhythm and with previously diagnosed AF, the SRA algorithm predicts AF with high specificity (99%) but relatively low sensitivity (60%) (12, 13).

Our study is the first to evaluate the usefulness of SRA in ambulatory monitoring of patients with undetermined TIA/RISS, showing high sensitivity (94%) and negative predictive value (98%) with relatively low specificity (78%). These results would support the discharge of patients with a normal result after a 2-h follow-up in the emergency room, without the need for hospitalization. For patients with an undetermined TIA and no evidence of AF on 24-h cardiac monitoring, ambulatory cardiac monitoring is recommended (21). According to previous studies, the rate of AF detection in the early hours and days after stroke is much higher compared to later periods (14).

However, several important questions still need to be determined: How much monitoring time is necessary? How often should monitoring be repeated? And for how long should this monitoring be prolonged? In our study, we detected AF in nine cases (47.4% of all FA) in the first 7 days of monitoring, six cases (31.6%) in the following 31–90 days, and four cases (21%) between 91 and 210 days from the onset of symptoms. According to our findings, about 50% of AF cases were detected in the first month, almost 80% in the first 3 months, and none beyond 7 months, which suggests that monitoring efforts should focus on this period.

**Figure 3:**
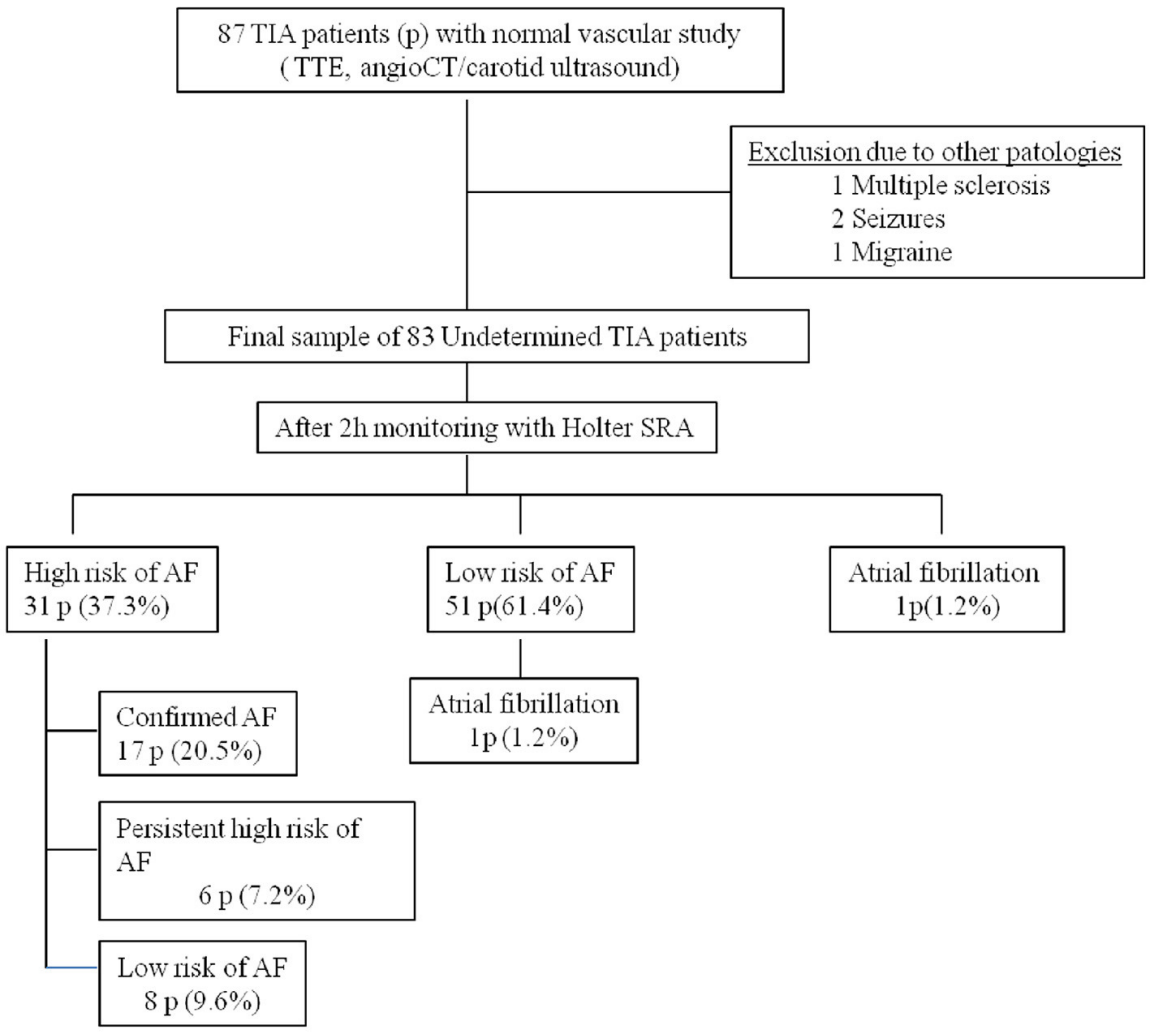
Results of the Holter-SRA analysis in our study population.

Our results support the hypothesis that early treatment and diagnosis can modify the natural history of TIA/RISS patients and reduce the risk of stroke recurrence. Three patients (3.6%) suffered a vascular recurrence at 90 days, a percentage lower than that reported in the literature (8–20%) (2–5), and this recurrence was due to unknown AF in all cases. Perhaps a more intensive monitoring protocol in the first month (weekly) could have decreased vascular recurrences by allowing earlier diagnosis. It is known that the risk of early recurrent stroke is related to various clinical factors such as age, diabetes mellitus, symptoms lasting over 10 min, weakness, impaired speech, etiological cause (22), as well as DWI abnormalities (22). For instance, TIA patients with an ABCD^2^ score of 4–5 points have a stroke risk of 9.8% at 90 days, while those with an ABCD^2^ score of ≥6 points have a risk of 17.8% at the same time point (23). In addition, the association of duration > 60 min with DWI abnormality is an independent predictor of further cerebral ischemic events (HR, 5.02) (22). In our study, 38.5% of patients presented an ABCD^2^ score ≥ 5 points, 85% had vascular risk factors, and 18% had acute lesions on DWI. Therefore, the low recurrence of stroke detected in our study is unlikely to be due to the characteristics of the sample. Previous studies in TIA patients evaluated with the gold standard (CEM) have reported an incidence of AF of 8% (14). In our study, we have increased the detection of AF to 22.9%. Therefore, we consider that Holter-SRA monitoring could be a more cost-efficient diagnostic strategy than hospital admission (Figure 3).

Although Holter-SRA monitoring presents a sensitivity of 78.4–100%, specificity of 86.2–95.7%, PPV of 16.5–90.6% and NPV of 96.2–100% for the detection of AF compared to the gold standard (CEM) (13, 14, 19) it cannot replace a manual evaluation because the proportion of false-positive patients with AF after SRA analysis is about 4–14%. In our study, the manual analyses reviewed by a cardiologist detected 5.5% false-positive results.

Our study has several limitations: (1) A large cohort would be necessary to evaluate the true risk of AF in TIA/RISS patients (2) Given the high negative predictive value of the Holter SRA in previous studies, prolonged monitorization was not performed in the low risk pattern group, which does not allow us to evaluate the sensitivity and specificity of the device in all patients. (3) Our study does not have a control group evaluated by CEM to compare the results of both devices, (4) We do not know the relative influence of each of the different treatments used on the risk of recurrent stroke. In our study, secondary prevention was initiated immediately, and all patients were evaluated by a stroke specialist in the emergency department and face-to-face at 90 days, with excellent adherence to prescribed medications, (5) Our study is limited to patients seen at a single institution, which may limit its generalizability to other patients populations, (6) Arterial stiffness was not analyzed, so we do not know if there were differences between patients with confirmed AF and those with a high or low risk pattern of AF.

In conclusion, Holter-SRA has enabled us to increase the detection of AF in patients with undetermined TIA/RISS, reducing vascular recurrences at 90 days. Prolonged monitoring of 7 days each month allowed us to detect ~80% of AF in the first 3 months and none beyond 7 months. Future studies may evaluate the design of the Holter-SRA diagnostic method on the incidence of AF.

## Data availability statement

The original contributions presented in the study are included in the article/supplementary material, further inquiries can be directed to the corresponding author.

## Ethics statement

The studies involving humans were approved by Code CEIC: 20201007—Department of Medicina Interna Center: Hospital General de Granollers Promotor: Hospital General de Granollers. The studies were conducted in accordance with the local legislation and institutional requirements. The participants provided their written informed consent to participate in this study.

## Author contributions

FC: Writing – original draft, Writing – review & editing. AI: Writing – original draft, Writing – review & editing. DC: Writing – original draft, Writing – review & editing.

## References

[1] Marini C De Santis F Sacco S Russo T Olivieri L Totaro R. Contribution of atrial fibrillation to incidence and outcome of ischemic stroke: results from a population-based study. Stroke. (2005) 36:1115–9. 10.1161/01.STR.0000166053.83476.4a 15879330

[2] Panuganti KK Tadi P Lui F. Transient Ischemic Attack. Treasure Island, FL: StatPearls (2023). 29083778

[3] Kleindorfer D Panagos P Pancioli A Khoury J Kissela B Woo D. Incidence and short-term prognosis of transient ischemic attack in a population-based study. Stroke. (2005) 36:720–3. 10.1161/01.STR.0000158917.59233.b7 15731465

[4] Hill MD Yiannakoulias N Jeerakathil T Tu JV Svenson LW Schopflocher DP. The high risk of stroke immediately after transient ischemic attack: a population-based study. Neurology. (2004) 62:2015–20. 10.1212/01.WNL.0000129482.70315.2F 15184607

[5] Amarenco P Lavallee PC Labreuche J Albers GW Bornstein NM Canhão P. One-year risk of stroke after transient ischemic attack or minor stroke. N Engl J Med. (2016) 374:1533–42. 10.1056/NEJMoa1412981 27096581

[6] Sanna T Diener HC Passman RS Di Lazzaro V Bernstein RA Morillo CA. Cryptogenic stroke and underlying atrial fibrillation. N Engl J Med. (2014) 370:2478–86. 10.1056/NEJMoa1313600 24963567

[7] Ntaios G Papavasileiou V Milionis H Makaritsis K Vemmou A Koroboki E. Embolic strokes of undetermined source in the athens stroke registry: an outcome analysis. Stroke. (2015) 46:2087–93. 10.1161/STROKEAHA.115.009334 26159795

[8] Kirchhof P Benussi S Kotecha D Ahlsson A Atar D Casadei B. 2016 ESC Guidelines for the management of atrial fibrillation developed in collaboration with EACTS. Eur Heart J. (2016) 37:2893–962. 10.1093/eurheartj/ehw210 27567408

[9] Sposato LA Cipriano LE Saposnik G Ruiz Vargas E Riccio PM Hachinski V. Diagnosis of atrial fibrillation after stroke and transient ischaemic attack: a systematic review and meta-analysis. Lancet Neurol. (2015) 14:377–87. 10.1016/S1474-4422(15)70027-X 25748102

[10] January CT Wann LS Alpert JS Calkins H Cigarroa JE Cleveland JC Jr. 2014 AHA/ACC/HRS guideline for the management of patients with atrial fibrillation: executive summary: a report of the American College of Cardiology/American Heart Association Task Force on practice guidelines and the Heart Rhythm Society. Circulation. (2014) 130:2071–104. 10.1161/CIR.0000000000000040 24682348

[11] Kernan WN Ovbiagele B Black HR Bravata DM Chimowitz MI Ezekowitz MD. Guidelines for the prevention of stroke in patients with stroke and transient ischemic attack: a guideline for healthcare professionals from the American Heart Association/American Stroke Association. Stroke. (2014) 45:2160–236. 10.1161/STR.0000000000000024 24788967

[12] Schaefer JR Leussler D Rosin L Pittrow D Hepp T. Improved detection of paroxysmal atrial fibrillation utilizing a software-assisted electrocardiogram approach. PLoS ONE. (2014) 9:e89328. 10.1371/journal.pone.0089328 24586692 PMC3938451

[13] Adami A Gentile C Hepp T Molon G Gigli GL Valente M. Electrocardiographic RR interval dynamic analysis to identify acute stroke patients at high risk for atrial fibrillation episodes during stroke unit admission. Transl Stroke Res. (2019) 10:273–8. 10.1007/s12975-018-0645-8 29971705 PMC6526141

[14] Gomis M Davalos A Purroy F. Stroke risk analysis, a system with a high detection rate of atrial fibrillation in stroke and transient ischemic attack. Stroke. (2020) 51:262–7. 10.1161/STROKEAHA.119.026354 31842722

[15] Adams HP Jr Bendixen BH Kappelle LJ Biller J Love BB Gordon DL. Classification of subtype of acute ischemic stroke. Definitions for use in a multicenter clinical trial. TOAST. Trial of Org 10172 in Acute Stroke Treatment. Stroke. (1993) 24:35–41. 10.1161/01.STR.24.1.35 7678184

[16] Rizos T Rasch C Jenetzky E Hametner C Kathoefer S Reinhardt R. Detection of paroxysmal atrial fibrillation in acute stroke patients. Cerebrovasc Dis. (2010) 30:410–7. 10.1159/000316885 20720410

[17] Rizos T Guntner J Jenetzky E Marquardt L Reichardt C Becker R. Continuous stroke unit electrocardiographic monitoring versus 24-hour Holter electrocardiography for detection of paroxysmal atrial fibrillation after stroke. Stroke. (2012) 43:2689–94. 10.1161/STROKEAHA.112.654954 22871678

[18] Reinke F Bettin M Ross LS Kochhäuser S Kleffner I Ritter M. Refinement of detecting atrial fibrillation in stroke patients: results from the TRACK-AF Study. Eur J Neurol. (2018) 25:631–6. 10.1111/ene.13538 29205690

[19] Rogalewski A Plümer J Feldmann T Oelschläger C Greeve I Kitsiou A. Detection of atrial fibrillation on stroke units: comparison of manual versus automatic analysis of continuous telemetry. Cerebrovasc Dis. (2020) 49:647–55. 10.1159/000511563 33207338

[20] Joglar JA Chung MK Armbruster AL. 2023 ACC/AHA/ACCP/HRS guideline for the diagnosis and management of atrial fibrillation: a report of the American College of Cardiology/American Heart Association Joint Committee on Clinical Practice Guidelines. Circulation. (2024) 149:e1–156. 10.1161/CIR.0000000000001193 38033089 PMC11095842

[21] Kleindorfer DO Towfighi A Chaturvedi S Cockroft KM Gutierrez J Lombardi-Hill D. 2021 guideline for the prevention of stroke in patients with stroke and transient ischemic attack: A Guideline From the American Heart Association/American Stroke Association. Stroke. (2021) 52:e364–467. 10.1161/STR.0000000000000375 34024117

[22] Purroy F Montaner J Rovira A Delgado P Quintana M Alvarez-Sabin J. Higher risk of further vascular events among transient ischemic attack patients with diffusion-weighted imaging acute ischemic lesions. Stroke. (2004) 35:2313–9. 10.1161/01.STR.0000141703.21173.91 15322305

[23] Wasserman J Perry J Dowlatshahi D Stotts G Stiell I Sutherland J. Stratified, urgent care for transient ischemic attack results in low stroke rates. Stroke. (2010) 41:2601–5. 10.1161/STROKEAHA.110.586842 20947856

